# Simulated Amaurosis

**Published:** 1873

**Authors:** George C. Harlan

**Affiliations:** Surgeon to Wills (Ophthalmic) Hospital, Philadelphia


### SIMULATED AMAUROSIS.

BY GEORGE C. HARLAN, M.D.,

*Surgeon to Wills (Ophthalmic) Hospital, Philadelphia.*

Two years ago a girl eleven years of age, apparently in excellent health was brought to me by her parents, with the statement that they had recently discovered her left eye to be quite blind. She had been sent to the family physician, a homœopath, on account of a slight conjunctivitis, and, as the result of the consultation, had returned with this startling announcement. She denied even the perception of light in that eye.

Never having met with a similar case, I failed to detect, or even suspect, the deception, but as a careful ophthalmoscopic examination revealed no lesion or imperfection of the organ, I assured the girl's parents that there was no present disease there, and no indication for treatment. The other eye was found to be perfect in all respects, and I advised them to return her to school and pay no attention to the blind eye while the sight of the other remained unimpaired.

A few weeks since, she was again brought to my office complaining that the other eye was failing, the left still continuing stone blind. She said that the print looked blurred when she attempted to read, and her parents had noticed that she held the book nearer to the eye than usual.

She admitted a vision of $= \frac{20}{L}$ and with a— $\frac{\text{[illegible]}}{18}$ glass acknowledged to $\frac{20}{XL}$. A central limitation of the field of vision was very well counterfeited. The result of a careful ophthalmoscopic examination was entirely negative. While I was engaged in recording her case, she amused herself by looking over the glasses in the trial case, and announced that the plain blue glass made a great improvement in her sight. This being the only discoverable indication for treatment, I ordered blue glasses. Not finding the large coquilles, with which the optician furnished her becoming, she, at the next visit, denied that they were of any use. When directed to look at a distant gas-light through a prism, with its base upward, held before the right eye, she at once acknowledged the double images, but when the attempt was made to separate the images by means of a colored glass her suspicions seemed to be aroused, and her answers were negative. When required to read the test letters at varying distances there were evident discrepancies in her answers.

The pretended amblyopia of the right eye precluded the use of any of the tests based upon the reading of ordinary type, and confined me to the large letters which she had acknowledged being able to recognize. I, therefore, placed the trial frames before her eyes, with a plain glass in the left side, and a convex one of ten inches focal distance in the right. The latter, without exciting her suspicion, excluded the right eye from any distinct vision beyond the focus of the glass. She still read No. L at twenty feet. Then having first substituted for the test card another with a different series of letters, I placed an opaque disk in front of the plain glass and she could not make out a letter. This proved at least that she had been reading No. L at twenty feet with the eye that she pretended was not conscious even of the bright glare from the ophthalmoscopic mirror, and was quite enough to throw a degree of doubt, almost amounting to certainty, on all her statements.

She did not appear again, but her father called on me some days afterward, and reported that she had come home in a very bad humor, indignantly accusing me of having treated her with great injustice, but, the next day, had made admissions, which, though partial and constrained, were sufficient to convince him fully of the correctness of my view of her case.

The second case was seen in consultation with Dr. Goodman, and has beon reported in the *Philadelphia Medical Times* for August 15, 1872.

A rather delicate looking boy, æt. 11, was represented to have become blind in the right eye about eighteen months before. After the disease had resisted the skill of the family physician for some months, the patient's father was recommended by his friends to "try electricity," and took him to a "professor" of that science. The result of one application was an entire restoration of vision. The cure lasted, however, only a few weeks, when the remedy was resorted to again with the same result as before. After this history had repeated itself a number of times, the father, as a measure of economy, invested in an apparatus of his own, and wound up his son's vision as often as it ran down.

When brought to us, the boy asserted that the eye was not conscious of the ophthalmoscopic examination, which, however, revealed nothing to account for the loss of sight. When a prism was placed before the left eye, he admitted seeing two images of the gas jet which could easily be distinguished by a colored glass, or made to unite by a corrective squint, when the base of the prism was held outward. He was required to read with the prism still before the eye, and while he was disconcerted and thrown off his guard by being urged to read rapidly, a fold of paper was slipped in front of the left eye, and he continued to read with the right.

The father was quite sure that there was no motive for the deception. The fact that the boy's mother was blind of the right eye was a singular coincidence and perhaps had a psychological bearing on the case.

Though this form of deception has been occasionally met with among the strange vagaries of hysterical women, and has often been resorted to by men with some object in view, it is extremely uncommon in boys. I know of but one other case. It is reported by Galezowski in his recent work on diseases of the eye.

The patient, or rather the culprit, was a boy of eleven years, and Galezowski considered it a plain case of malingering to avoid school. In this the young scamp was quite successful for a length of time, until he was finally brought to Paris for treatment, and exposed by the prism test.

In the notes of the third case I am indebted to Prof. D. H. Agnew.

"Miss M. æt. 18 years, had always enjoyed excellent health. One year before my visit she was thrown from a horse. A short time after this accident, a deep-seated soreness was experienced in the right iliac region. Five months afterward a marked swelling made its appearance in the same locality, which disabled her from taking exercise, and very soon confined her to bed. At the time of my visit I found a conical enlargement, hard, sensitive, not moveable, and quite the size of the closed hand. The patient was somewhat pale, passed restless nights, with febrile disturbance toward evening, but no chills. The menstrual discharge was regular, only somewhat profuse, and attended by pain for the first day or two. A vaginal examination revealed an undue fulness on the right wall of the canal, high up at its insertion on the uterus. Her appetite was capricious, and her mental state somewhat depressed, though by no means despondent.

"I, diagnosed a periuterine abscess, depending, doubtless, upon a cellulitis set up by the violent concussion to which the pelvic viscera had been subjected by the fall.

"After the lapse of about four months the abscess opened into the vagina, the external swelling subsided, and finally the discharge disappeared and the patient was apparently well. About this time a tooth which gave her a great deal of pain was extracted by her dentist, while she was under the influence of chloroform, administered by myself. She recovered quietly and fully from the anæsthetic, and without any succeeding ex citemant whatever; in fect, her whole deportment, both before and after its exhibition, was cool and composed. The patient was now able to walk out, and was recovering her strength rapidly under a course of tonic treatment.

"Some weeks after this I was summoned to see her again. She had, in the course of the night, become entirely blind. Nothing had occurred the previous day to disturb either her mental or physical state. I watched her behavior very narrowly, and certainly every movement betokened a freedom from all attempts at acting a part; and indeed, the uniform courage with which she had endured her protracted suffering, her strong common sense, and pure character, all conspired to convince me that there was no dissimulation in the case.

"The pupil responded promptly and naturally to light, the external appearance of the eyes was normal, and an ophthalmoscopic examination failed to detect any morbid change.

"Believing the case to be one of hysterical blindness, I so stated to her friends, predicting that her vision would be recovered—how soon I did not venture to affirm. She was advised to continue the tonic treatment and use the shower bath.

"For some time she was led by her sister, until her familiarity with the house became such that she could move without assistance. Often did I watch her when entirely ignorant of my presence. With elevated head, open eyes, advanced and oscillating arms, the feet sliding along, would she thread her way about the room until the object which brought her in had been obtained. So convinced was I of the real nature of the delusion, if it may be so called, that I resorted to no test to detect simulated blindness.

"After she had been in this condition four weeks, the spell was dissolved as suddenly as it came; and her vision, which disappeared in a night, returned in a night."

No motive for deception could be discovered in any of these cases, and in the absence of other explanation, we are forced to fall back upon the term "hysterical," which is often another word for inexplicable, or they may be classed among the cases "which indubitably show that the simulation of disease has frequently been practised without the existence of any interested motive, indeed, without motive of any kind; that there is, in short, a species of moral insanity of which this simulation is the characteristic." This view seems to apply particularly to the case reported by Dr. Agnew, who had enjoyed unusual opportunities of becoming famiiiar with the previous character and disposition of hispatient, and watched her with a great deal of care. Is it possible that she could have been psycologically blind—in a kind of visual trance—that the act of vision was carried as far as anatomy and physiology could take it, but that the disordered mind refused to receive its impressions, and that she really could not or did not see? In the use of monocular optical instruments, we can easily, after a little practice, disregard and suppress the images on the retina of the eye not in use, and in strabismus when, to avoid diplopia, the images of the eye have been neglected, it becomes amblyopic without disease. Such facts show thät it is possible for images correctly formed upon the retina not to result in vision, though the eye and nerve and brain may be healthy, and suggest the possibility of a mental suppression, as we often have a mental perversion, of vision. It is, after all, not more mysterious that the mind should disregard the images of real objects than that it should conjure up images of objects that have no existence.

Perhaps it may, in some cases at least, be more scientific as well as more just, to consider "hysterical blindness" a real mental disease, rather than the mere whimsical counterfeiting of a symptom.

It is probable that cases of simulated blindness are more frequent than the space accorded to the subject in the text-books of ophthalmic surgery, some of which do not even mention it, would lead us to suppose. Doubtless they might afford an explanation of a good many mysterious cases, and not a few wonderful cures.

Military surgeons have from time to time, given a good deal of attention to the simulation of blindness, with an evident motive, by malingerers. Probably the art of malingering was developed to its highest perfection among the French soldiers, by the ruthless and sweeping conscription of the First Empire, when even Frenchmen began to feel that "la Gloire" was too dearly purchased, and Fallot says: "There is no disease more frequently pretended than amaurosis by those who desire to withdraw from military service, and it is almost always the right one that is said to be affected." Though a good deal of ingenuity was devoted to the art of "old soldiering" during our late war, it does not seem to have taken this direction to any great extent. I can recall only one case, which was detected by Dr. Dyer, by means of the prism test, at the Satterlee Hospital, and reported by Drs. Keen, Mitchell, and Morehouse, in an article on malingering in the number of this Journal for October, 1864. It is not unlikely, however, that there may have been some cases of the kind among the fifteen hundred and twenty-two men reported on the sick list with "amaurosis," five hundred and fifty-six of whom were discharged. In the examination of a number of applicants for pension, with alleged injury or disease of the eyes, I have not met with any case of feigned blindness, but several have attempted to take advantage of errors of refraction by attributing their defective vision to some disease or exposure encountered in the service.

A number of tests have been proposed for the detection of feigned blindness.

Gavin, in his work on *Feigned Diseases*, published in 184[illegible], has a very interesting chapter on amaurosis. Though no one of the means of detection in use at that time was at all certain in itself, and few of them would be worth resorting to now, he shows that experienced surgeons managed to reach a much greater degree of precision than most of us would be likely to attain to, if deprived of our test-types, ophthalmoscopes, prisms, stereoscopes, etc. The use of mydriatics seems to have been quite common among army malingerers. The story is told of a

French conscript who had dilated his pupils widely with belladonna, and was so determined in his deception that when a sharp instrument was approached to the eye, as if to be plunged through the cornea, neither head nor eye stirred, but the palpitation of his heart betrayed him.

When one pupil only is dilated, the use of belladonna may be detected by the refusal of the pupil to act in unison with that of the other eye, which it does almost invariably in true amaurosis. This, of course, presupposes the integrity of the third pair of nerves, for cases of mydriasis, from paralysis of the sphyncter pupillæ, are frequently met with in which distant vision, at least, is unaffected. I have recently had under treatment at the Wills Hospital three or four cases which presented no other symptoms than mydriasis and paralysis of the accommodation, and which differed in no respect, except in persistence, from the action of atropia. It has been suggested, in cases of doubt, to puncture the anterior chamber and test the aqueous humor for the presence of atropia by applying it to a healthy eye.

Snellen's test types afford a simple and valuable means of detection when a diminution only, and not an entire absence of, vision is pretended. If No. XL, for instance, can be seen at ten feet, No. LXXX should be seen at twenty feet, and No. XX at five feet. The great frequency of errors of refraction must, however, be taken into account. A myopic eye might very well be able to distinguish No. XX at five feet, and fail to read No. LXXX at twenty feet, or the near vision of an hypermetropic eye might not correspond to the acuteness of its distant vision, while astigmatism would account for a much greater degree of amblyopia and a greater amount of seeming contradiction in the answers of the person examined.

For simulated monocular blindness, Graefe's prism test is a very convenient and useful one. If a prism, held before the eye in which sight is admitted, causes double vision, or, when its axis is held horizontally, a corrective squint, vision with both eyes is rendered certain. It must be remembered, however, that a useful degree of vision is not thus proved; it is only shown that the eye can see, but how much it can see must be determined by other tests. It should also be borne in mind that the failure to produce double images is not positive proof of monocular blindness, for it is possible that the person may see with either eye separately, but not enjoy binocular vision, as in a case of squint, however slight.

A test recently suggested by Warlomont is so absurdly simple that it seems almost inconceivable that no one should have thought of it before, but it has proved conclusive, even as legal evidence. Displace the optic axis by slight pressure with the finger upon the eyeball, and show the suspected person two small dots on a piece of paper; if he says there are four, he is at once convicted.

Javal's test is almost equally simple. Cause the person to read while a ruler is held three or four inches from the face and directly in front of the nose, then close the blind eye, and, as part of the print will be concealed from the other one by the ruler, it will be easily shown that he has been seeing with both.

The stereoscope affords a very elegant means of detection. If the two fields are united, binocular vision is, of course, proved. We may even test each eye separately, independently of simultaneous vision by both, as recommended by Schwiegger. (*N. Y. Med. Journal*, February, 1866.)

"If we draw in each separate field of vision that vertical line whose image goes through the centre of the retina, then, in the united stereoscopical field, not only both lines are seen as one, but every object situated on the right of one of these lines is projected to the right side of the field of vision and appears as if it were seen by the right eye. The same, of course, is the case with the left side. This gives the means of determining, in similar monocular amblyopia, the acuteness of vision, and, if we choose, even the range of accommodation. For this purpose, we arrange matters as follows: we have at the bottom of the stereoscope a sheet of paper marked only with the two lines above mentioned; now, if we have to deal, for instance, with an alleged amblyopia of the left eye, we place in the left field of the stereoscope, but to the right side of the vertical line, any object, say a piece of printed paper; with this exception, the whole of the bottom of the stereoscope is left blank. In the united stereoscopical field the paper will then appear as on the right side, and will make so strong an impression that it is seen with the right eye, that I doubt whether anybody can resist it. With a stereoscope which allows the convex lenses to be approached to, or withdrawn from, the bottom of the stereoscope, we can, if we choose, at the same time, ascertain the range of accommodation."

This plan has the advantage over that recommended by Laurence, by means of optical transposition of words upon the stereoscopic side, in the fact that it renders us independent of binocular vision.

When blindness of one eye only is alleged, if the different tests are skilfully and perseveringly varied and combined, a very considerable knowledge of optics, more than is likely to be devoted to such a cause, would be required to escape conviction.

Fortunately, the simulation of complete blindness of both eyes involves so many inconveniences, that it is not often resorted to. No optical test can, of course, be applied to it. Patient waiting and careful watching will usually discover such a case. Dr. Hutchinson's success in speedily and permanently curing a case of deaf dumbness by means of etherization (*Am. Journ. Med. Sci.*, April, 1864) might suggest a trial of that means. As the effect of the anæsthetic passed off, the patient would probably recover the power of vision before his consciousness was sufficiently restored to enable him to resume the deception.

This plan was recommended in the article on malingering, referred to above, but I am not aware that it has ever been practiced.—*American Journal of the Medical Sciences.*

